# Class-room Talks, No. 3

**Published:** 1888-03

**Authors:** J. T. Kent


### HOMŒOPATHIC PHYSICIAN,

A MONTHLY JOURNAL OF MEDICAL SCIENCE.

"If our school ever gives up the strict inductive method of Hahnemann, we are lost, and deserve only to be mentioned as a caricature in the history of medicine."—CONSTANTINE HERING.

Vol. VIII. MARCH, 1888. No. 3.

### CLASS-ROOM TALKS. No. 3.

(FROM THE LECTURES OF PROF. J. T. KENT, M. D.)

How do we tell the symptoms of sickness in babies? Why, every motion baby makes means *something*. You are supposed to know how a healthy baby looks and acts, that all the funny wrinkles and twists he makes, waking and sleeping, are not sickness, just as you are supposed to know the healthy physiological from the unhealthy pathological conditions existing in the adult. When Master Baby continues to cry and cry, unsoothed by any of the usual methods of supplying his wants, something is wrong, and we study him well to find what it may be.

How do we know when baby has earache? He will cry incessantly, and if you are observant you will find he tries awkwardly to put his hand to his ear. Possibly he will wish to be carried constantly, like Cham. and Puls. These remedies usually cover most cases of earache in babies. The Pulsatilla baby will wish to be carried *slowly*. The constant cry will be sad and heart-broken. The Chamomilla baby will need to be carried more rapidly, will show temper and scream lustily. An observant nurse will tell you that he is easier when pressing the affected side to her shoulder while carrying him. Therefore you will say it is better from *heat applied*. Now, what shall we give? Cham.? Right. If he is *agg.* by the warmth gathered by lying upon her breast, what would be the remedy? Puls. Now, suppose the little fellow is good in the open air; is good so long as nurse keeps him out-of-doors, gently rolling him about? Puls. How may we know when baby has retention of urine? Simply enough—a continual clawing at the genitals, with dry napkins. Suppose baby is constantly pulling at the scrotum, what symptom would you suppose to be present? Formication. What remedy has this symptom? Staph. An adult expresses to you by words what baby can only tell by signs. You must learn to read those signs. One more symptom of this sign language and we will leave the chair of pædology, upon which we are trespassing—baby is continually "elongating the penis." What remedy? Merc. We have most torturing pains arising from suppressed ear-discharges. I have had under my care the past three years the case of a woman who has suffered from an ear-discharge since childhood. She *had* tried all the doctors of all the "paths" without a cure. Each had given promise of speedy relief, and had begun with local treatment, always with the result of suppressing the discharge, but bringing on a most frantic neuralgia of scalp and head. She has been a terrible sufferer. Has had necrosis of the bones of the ear, with watery, briny, sanious, offensive discharge. Since beginning treatment homœopathically the character of the discharge has improved, she suffers less than in years before, has much more comfort, and in three years more I hope to have her in so healthy a condition that her life will be tolerable to herself and her friends.

In all discharges it is nature's effort to rid herself of abnormal conditions, or, better still, to make such expression of the sick-making power within as may lead you to her relief with an appropriate remedy selected according to the only law of healing. Check this expression, and you may bring about the most terrible suffering, if not *death*. If you are an ignoramus you will call the new suffering another disease.

You will be called upon to treat certain conditions that arise, in themselves *unpleasant*, yet not actually *painful;* and let me warn you now that if you suppress without homœopathically curing, and have sufficient brain to see the results of your interference, you will curse the day you ever gave a dose of medicine. From Silicea—of which drug I have read you the manner of preparation for homœopathic use—vegetation, grain, corn, etc., and inanimate nature, as in the rocks and sands, receive that shining smoothness, the glazing, as of the cane, which, like a varnish, protects them from the destructive action of heat and moisture and adds strength, toughness, and elasticity to their fibre, giving to the plants their uprightness and resistance to wind and storms, those qualities without which they would be short-lived and useless.

In the human family we find the analogy perfect. The toughness and elasticity of fibre, the external polish and softness of finish in both hard and soft structures—*all* are there. Without Silicea the lime in the bones would be but a friable mass, the hair would lose its glossiness and fineness, the skin its softness and polish, the nails their toughness and smoothness, the mind its strength to do and dare. The whole physical world would fall into distress and decay, uncomfortable beyond belief. Silicea is a necessity to both plant and animal life, and an inability to assimilate it creates an inanition for it, such as we find in provers of the drug. We see the need for Silicea in the similarity of its symptoms to such a condition in such provers and in sick humanity. We find great mental weakness, great physical weakness, a general loss of tone and healthiness of the whole system. This mental debility of the patient leads to fear and shrinking from his ordinary duties, a dread of undertaking new projects, a fear of failing in his customary field of labor, especially when it involves an appearance before many people; yet, place the patient in a position in which he could no longer avoid or excuse himself from accepting, and he nerves himself to action, going through the work gloriously. Silicea corresponds to mental conditions found among men of public life, lawyers, bankers, men of affairs whose mind has long been kept tense and unyielding, until at last nature gives way, and brain-fag, preceding softening, is often the result.

Within four-and-twenty hours I have been paralyzed with astonishment at a question put to me by a homœopathic (?) doctor. "Where," said he, "did you find *your* idea of teaching the red-string of the remedy?" Not know the source from which I obtained the drug-picture, the "red-string" that I taught daily in college! Where had he been? What had he read, not to know that Hahnemann's *Chronic Diseases* contains the most beautiful word-pictures of drugs that have ever been written or spoken? *I* cannot do *as well.* I wish I could—could give such glowing pictures of each drug in its effects and modalities as Hahnemann, with his clear perceptions of the drug in its beautiful entirety. A homœopathic doctor to ask a question like that! Why, Bœnninghausen named it the "red string" years ago, named the drug-picture that Hahnemann wrote out. The sailors on shipboard learned to know good English rope by the interwoven "red-string" which extended its length. The "red-string" gave character to the rope, and it was reliable rope. I suspect it is much so with the remedies; the ones in which you see the "red-string" prominently will prove to you reliable.

No worse homœopath ever existed than I when I began its study and practice. I had to begin at the beginning, as you will have to do, and had to put to myself the question of the practice I should follow—whether it should be the old induction method of Hahnemann or that of the so-called *modern* homœopaths and *physiological humbugs.* Hahnemann, as you know, was not the first to mention or discover this existing law of similars; but he did discover the only possible means of applying this *law*, by proving the action of drugs upon the healthy organism, by the dynamization of the drug and the administration of it to the similarly sick, where it became a most efficient remedy. This had occurred to no one. Hahnemann, by his indefatigable industry, proved and wrote out in detail a great number of drugs, doing all more clearly and perfectly than any have been able to do since. I studied both sides of the question, proved Hahnemann's method correct, and to the best of my understanding have followed the law. You may take the same well trodden pathway, but your progress will be greater and you will accomplish more if you begin where Hahnemann ended; for Homœopathy is but in its infancy, and we hardly know the first letters of its alphabet. S. L. G. L.

